# A Ten‐Country Study on Public Perceptions of 5G EMF Emissions: Who Feels Exposed, and Why?

**DOI:** 10.1002/bem.70058

**Published:** 2026-06-05

**Authors:** Sarah C. Link, James Grellier, Leanne Martin, Marie Eggeling‐Böcker, Ferdinand Abacioglu, Carolin Schulz, Nina Vaupotič, Mathew P. White, Christoph Boehmert

**Affiliations:** ^1^ TU Wien Vienna Austria; ^2^ Department for Social Sciences IU International University of Applied Sciences Erfurt Germany; ^3^ Public Health and Sport Sciences, University of Exeter Medical School University of Exeter Penryn Cornwall UK; ^4^ Department of Cognition, Emotion, and Methods in Psychology University of Vienna Vienna Austria; ^5^ Department of Clinical and Health Psychology University of Vienna Vienna Austria; ^6^ Aalen University Aalen Germany

**Keywords:** electromagnetic fields, exposure assessment, exposure perception, health risk perception, mobile communications

## Abstract

Formal risk assessment considers characteristics such as proximity, dose, and vulnerability. However, public risk perception may also be influenced by other—possibly less relevant—factors such as visibility and novelty. The introduction of 5G and its associated infrastructure and radiofrequency electromagnetic fields (RF‐EMF) may therefore change perceptions of RF‐EMF from mobile communications in general. To explore this, we conducted an online survey in 10 European countries (*n* = 10,358) using a picture‐based approach. Respondents perceived daily RF‐EMF exposures as moderate but expected them to increase with 5G. A mobile phone at the ear was generally associated with higher perceived exposure than multiple base stations. Overall, distance to the RF‐EMF source most strongly influenced perceived exposure, followed by the number of sources. 5G reception was linked to higher exposure perception than 4G or Wi‐Fi reception. These patterns were consistent across most countries. We conclude that when assessing RF‐EMF exposure, people rely on heuristics (e.g., more sources imply more exposure) that often guide them correctly. Understanding when and why people feel particularly exposed can help develop more effective communication about true levels of exposure and risk.

## Summary

- Exposure to RF‐EMF is perceived as moderate but expected to increase due to 5G.
- The exposure characteristic distance influences exposure perception most.
- Significant differences between the 10 countries studied.

**Abbreviations**
5G: fifth generation of mobile technologies
EMF: electromagnetic fields
GHz: gigahertz
IARC: International Agency for Research on Cancer
ICNIRP: International Commission for Non‐Ionizing Radiation Protection
kHz: kilohertz
RF‐EMF: radiofrequency electromagnetic fields
WHO: World Health Organization

## 1 Introduction

Humans lack the ability to sense radiofrequency electromagnetic fields (RF‐EMF), which are used in mobile communications to transmit data such as voice or text. Double‐blind provocation studies have shown that even individuals claiming to be RF‐EMF sensitive cannot perceive true exposure (Bosch‐Capblanch et al. 2024; Röösli et al. 2010; Rubin et al. 2010). Lacking adequate measurement tools, people apply other criteria to estimate exposure.

RF‐EMF are characterized as non‐ionizing radiation with frequencies from 100 kilohertz (kHz) to 300 gigahertz (GHz) that propagate at the speed of light. They occur naturally and can be modulated to transmit information, enabling mobile communication between mobile phone base stations (or “base stations” for short) and mobile phones. Both infrastructure elements act as transmitters and receivers.

Since 2019, the fifth‐generation mobile communications standard (5G) has been rolled out across Europe, with network coverage reaching 89% by late 2023, the time when data for the present study were collected (European Commission 2024). The timing and extent of the rollout differed between countries, which may have resulted in variations in exposure patterns and public familiarity with the technology across Europe.

International radiation protection authorities periodically assess possible adverse health effects of RF‐EMF and issue recommendations to protect the public. These are applied or adapted by national radiation protection agencies (Missling et al. 2016). So far, there is scientific evidence of thermal effects (ICNIRP 2020), referring to tissue heating due to exposure. To prevent possible negative health effects due to heating, exposure limits are applied, below which no negative health effects have been proven (ICNIRP 2020). However, the International Agency for Research on Cancer (IARC) classified RF‐EMF as “possibly carcinogenic to humans” (Group 2B) (IARC 2013), indicating limited or insufficient evidence for a carcinogenic effect (IARC 2013). To obtain more scientific evidence on the effect of RF‐EMF, the World Health Organization (WHO) commissioned 10 systematic reviews (Verbeek et al. 2021). By now, both the systematic reviews and a contextual publication (Verbeek et al. 2025) have been released. The latter concisely summarizes the main findings: nearly all reviews concluded that RF‐EMF has no or only small effect. Certainty of the evidence was very low to moderate for observational studies and moderate to high for experimental studies. Evidence from animal studies indicated a high‐certainty effect on male fertility. For outcomes such as oxidative stress, thyroid cancer, or human fertility, the available evidence remains limited or inconsistent (Verbeek et al. 2025). To date, neither ICNIRP nor IARC has updated its risk assessment based on these systematic reviews.

Despite these assessments, public concern about potential adverse (health) effects persists (Frey 2021; TNS Opinion & Social 2010). The “intuitive toxicology” framework (Kraus et al. 1992) explains such responses: people perceive potentially toxic substances on different sensory levels, which give them clues as to whether something is dangerous or not. However, the assessment of laypeople and experts often diverges. Laypeople may perceive natural hazards as less dangerous than artificial ones and underestimate the role of dose (Kraus et al. 1992). Studies confirm limited public understanding of toxicological principles, which is often associated with a rejection of chemicals (Bearth et al. 2019). Though originally applied to chemicals, intuitive toxicology has also been linked to RF‐EMF (Freudenstein, Wiedemann, and Varsier 2015b).

Risk perception of RF‐EMF varies by sociodemographic factors. Women generally show a higher risk perception than men (Dilkova‐Gnoyke et al. 2022; Eggeling‐Böcker et al. 2025; Frey 2021; Siegrist et al. 2005). Evidence on age effects is mixed: some studies found no influence (Frey 2021; Siegrist et al. 2005), whereas age was positively related to concern in another study (Dilkova‐Gnoyke et al. 2022). Recent research has not yet addressed the role of education in RF‐EMF risk perception.

### 1.1 Exposure Perception and Risk Perception

Exposure perception is closely related to risk perception (Freudenstein, Wiedemann, and Brown 2015). Recent literature characterizes risk perception as a multidimensional construct (Walpole and Wilson 2021), with perceived exposure forming one component. However, since risk only arises under exposure, perception of exposure is a necessary element of perceived risk (e.g., Aven 2012).

Exposure perception refers to the degree to which individuals believe they are exposed to an agent in a given context (Link et al. 2025). It has been investigated in relation to RF‐EMF (Freudenstein, Wiedemann, and Brown 2015; Freudenstein, Wiedemann, and Varsier 2015; Link et al. 2025), but studies have typically reported on the influence of different exposure characteristics (e.g., duration) on exposure perception as secondary findings. Freudenstein, Wiedemann, and Varsier (2015) found that people evaluate the impact of duration, strength, distance, frequency, and number of exposure sources on EMF health risks similarly (pre‐5G).

The effect of the source of exposure on risk perception has been extensively studied. Evidence consistently indicates that individuals overestimate RF‐EMF exposure from base stations relative to mobile phones (Cousin and Siegrist 2010b; Freudenstein, Wiedemann, and Brown 2015), especially when it comes to the perception of risk for oneself (White et al. 2007). Yet, given the close proximity of handsets to the body, mobile phones are usually the most relevant source of exposure. Recent data suggest increasing public awareness of this relationship (Link et al. 2025; Vaupotič et al. 2025).

Still, distance strongly shapes perception: individuals prefer locations farther away, assuming lower exposure (Cousin et al. 2011; Cousin and Siegrist 2010a, 2010b). Evidence on the influence of the number of sources, particularly base stations, is mixed (Claassen et al. 2014; Cousin and Siegrist 2010b, 2010a; Freudenstein, Wiedemann, and Varsier 2015).

### 1.2 5G Exposure

With the introduction of 5G, exposure has changed compared to legacy networks, presenting new and more complex exposure patterns and characteristics. For instance, in the future 5G will use frequency bands with shorter wavelengths and therefore shorter transmission distances, requiring denser base station networks (Schmidt 2020). In urban areas with denser networks, downlink exposure becomes more relevant than uplink exposure (CLUE‐H 2026). Thus, it is difficult to draw general conclusions about how true exposure has already changed and will change in the future due to 5G and the fact that new frequencies, exposure scenarios, and settings are introduced (CLUE‐H 2026).

### 1.3 Exposure Perception of 5G

Due to these technical and exposure differences between 5G and earlier standards, we considered it particularly important to examine whether exposure to 5G is perceived differently. Existing studies on 5G perception have primarily focused on perceived risks (Dilkova‐Gnoyke et al. 2022; Frey 2021; Koh et al. 2020), or technology acceptance (Al‐Maroof et al. 2021). Public acceptance of 5G has also been impacted by misinformation and conspiracy theories making unfounded claims associated with COVID‐19 (Ahmed et al. 2020; Cinelli et al. 2020).

Link et al. (2025) conducted a qualitative, picture‐based (interview) study with a focus on 5G. Participants were presented with a variety of exposure situations that differed systematically in terms of RF‐EMF source, network type, number of RF‐EMF sources, proximity of RF‐EMF sources to the body, and the direction of data transfer. Participants associated 5G with higher perceived exposure than 4G, especially for base stations, but sometimes also for mobile phones. A larger number of sources—particularly base stations—was linked to higher perceived exposure, though participants were uncertain whether multiple distant phones implied more exposure than one nearby phone. Scenarios involving proximity to sensitive body parts (e.g., head or reproductive organs) also yielded higher perceived exposure.

Although insightful, the number of participants in this study was relatively small and we do not know how generalizable these conclusions are to more representative samples or people across different country contexts. The present study therefore focusses on the exposure characteristics *network type, number of exposure sources*, *proximity/distance*, and *data transfer*.

### 1.4 Research Questions and Hypotheses

Building on prior research, this study addressed three research questions (RQ) with corresponding hypotheses.

**RQ1:** How do people perceive exposure to RF‐EMF emitted by 5G?

Hypothesis 1A On average, in the countries investigated, people perceive their everyday exposure to RF‐EMF differently.

Hypothesis 1B On average, in the countries investigated, people assume exposure to RF‐EMF will increase due to the introduction of 5G to a different degree.

**RQ2:** In which situations do people feel particularly exposed to RF‐EMF emitted by mobile communications?

Hypothesis 2 On average, people think that RF‐EMF emissions are higher from 5G than from 4G in terms of both mobile phone (A) handsets and (B) base stations.

Hypothesis 3 On average, people think that RF‐EMF emissions from mobile phone handsets are higher from 5G than from Wi‐Fi.

Hypothesis 4 On average, people associate a higher number of RF‐EMF sources with a higher exposure perception. This is the case for mobile phone (A) handsets and (B) base stations.

Hypothesis 5 On average, situations in which the mobile phone is closer to the body are associated with a higher exposure perception than situations in which the mobile phone is further away from the body.

Hypothesis 6 On average, people do not differentiate between upload and download activities regarding perceived exposure.

**RQ3:** Do people from different countries, age groups, genders and educational levels perceive exposure emitted by mobile communications differently?

This question was examined for the exposure characteristics described in Hypotheses 2–6.

Hypotheses and analysis methods were preregistered under https://osf.io/sm8ux/?view_only=2e8b73b8bf50407687c9a53b949699e8. Deviations from the preregistration are listed in Supporting Information S1: Appendix A.

## 2 Material and Methods

### 2.1 Data Collection

An online panel survey was conducted between September and December 2023 in 10 European countries differing in 5G network coverage (European Commission 2024) and possibly also in RF‐EMF risk perception (TNS Opinion & Social 2010): Austria, Finland, France, Germany, Greece, Poland, Serbia, Slovenia, Spain, and the United Kingdom. Respondents were recruited and incentivized via a panel provider. Individuals over the age of 16, who were part of the national panels, were eligible to participate.

Approximately 1000 respondents per country participated, representative for age, gender, and NUTS‐1 region (European Commission 2019). This sample size follows established practice in cross‐national survey research and represents a standard benchmark for obtaining reliable and comparable national‐level estimates, irrespective of population size. It further ensured that all quota requirements could be consistently fulfilled within each country. Interlocking quotas were used for age and gender, and non‐interlocking quotas for region. “Country affiliation” refers to panel membership, not nationality, which was not assessed. Given the large overall sample size (*n* ≈ 10,000), a sensitivity power analysis indicated that the study had 80% power (*α* = 0.05, two‐tailed) to detect very small effects (approximately *d* = 0.05 for mean differences, *r* = 0.03 for correlations, and *f* = 0.03 for ANOVA), demonstrating high sensitivity even to subtle differences.

### 2.2 Sample

Respondents who completed the survey unrealistically fast (< 10 min), too slowly (> 1 h), or failed either of two attention checks were excluded (recruitment and exclusions are summarized in Table 1). Thresholds were based on speed pre‐tests (*n* = 20), indicating a reasonable completion time of around 17.5 min. The mean completion time in the final sample was 21 min. Respondents received points from the panel provider as compensation. The final weighted dataset comprised 10,066 (unweighted 10,358) individuals (mean age = 48 years, range 16–93). Gender distribution was 51.1% female, 48.2% male, and 0.6% diverse or transgender. Table 2 shows the composition of the country samples in terms of age, gender, and education.

**Table 1:** Recruitment and exclusions of the respondents (total sample and by country).

	Total sample	Austria	Finland	France	Germany	Greece	Poland	Serbia	Slovenia	Spain	United Kingdom
Clicked on the survey link	30,647	3108	2669	3417	2810	2431	3113	2379	2465	3013	5242
Did not start participation	286	13	22	23	10	25	54	21	34	22	62
Speeding (< 10 min)	875	55	60	87	87	52	104	33	135	82	180
Inactivity (> 60 min or interrupted)	1883	142	163	194	139	155	262	186	231	180	231
Quota already full	10,698	1462	905	1528	1133	524	555	585	338	1009	2659
Failed attention checks	6547	415	501	566	412	640	1098	515	703	691	1006
Planned exclusions (sum)	20,289	2087	1651	2398	1781	1396	2073	1340	1441	1984	4138
Final sample	10,358	1021	1018	1019	1029	1035	1040	1039	1024	1029	1104

*Note:* Absolute (unweighted) numbers for the total sample and the country samples are reported.

**Table 2:** Sociodemographic composition of the sample.

	Total sample	Austria	Finland	France	Germany	Greece	Poland	Serbia	Slovenia	Spain	UK
*n*	10,066	1010	1009	1008	1007	1002	1002	1001	1011	1007	1009
Age											
*M* (SD)	48 (17.09)	47 (17.02)	48 (17.79)	48 (17.79)	49 (17.62)	49 (16.78)	47 (16.88)	48 (16.36)	48 (16.37)	48 (16.28)	47 (17.86)
Gender											
Men	48.2%	48.3%	48.5%	47.4%	48.6%	47.9%	47.7%	48.2%	48.8%	48.2%	48.5%
Women	51.1%	50.7%	50.6%	51.8%	50.7%	51.9%	52.1%	51.7%	50.1%	51.1%	50.6%
Other^a^	0.6%	1.00%	0.9%	0.8%	0.7%	0.2%	0.2%	0.1%	1.1%	0.7%	0.9%
Education^b^											
1	0.4%	0.1%	0.1%	0.8%	0.6%	0.4%	0.1%	0.1%	0.4%	0.5%	0.6%
2	11.2%	22.2%	9.7%	9.1%	19.2%	4.0%	7.3%	2.4%	3.6%	12.0%	22.4%
3	44.9%	53.1%	53.1%	36.1%	48.3%	29.6%	54.1%	44.3%	50.2%	39.0%	31.7%
4	43.5%	24.6%	37.1%	53.9%	31.9%	56.0%	38.5%	53.2%	45.8%	48.5%	45.3%

*Note:* Distribution based on the weighted data for the total sample and the country samples.

^a^ “Other” includes the categories “diverse,” “transgender,” and “other.”

^b^ Education: 1 = did not complete compulsory education, 2 = completed compulsory education, 3 = completed further education, 4 = completed university degree(s).

### 2.3 Exposure Perception Measures

Exposure perception was examined both generally and situation‐specifically. General exposure perception was assessed with two items.

1. *Everyday exposure perception*: “How much do you think you are exposed to electromagnetic fields (EMFs) from mobile communications devices and mobile phone masts (incl. 5G technology) in your everyday life?,” rated on a 7‐point rating scale (1 = not at all, 4 = moderately, 7 = to a very high degree).
2. *Perceived change in exposure:* “How much do you think people's exposure to electromagnetic fields (EMFs) changes due to the introduction of 5G?,” rated on a 7‐point Likert scale (1 = decreases very much, 4 = stays the same, 7 = increases very much).

**Figure 1:**
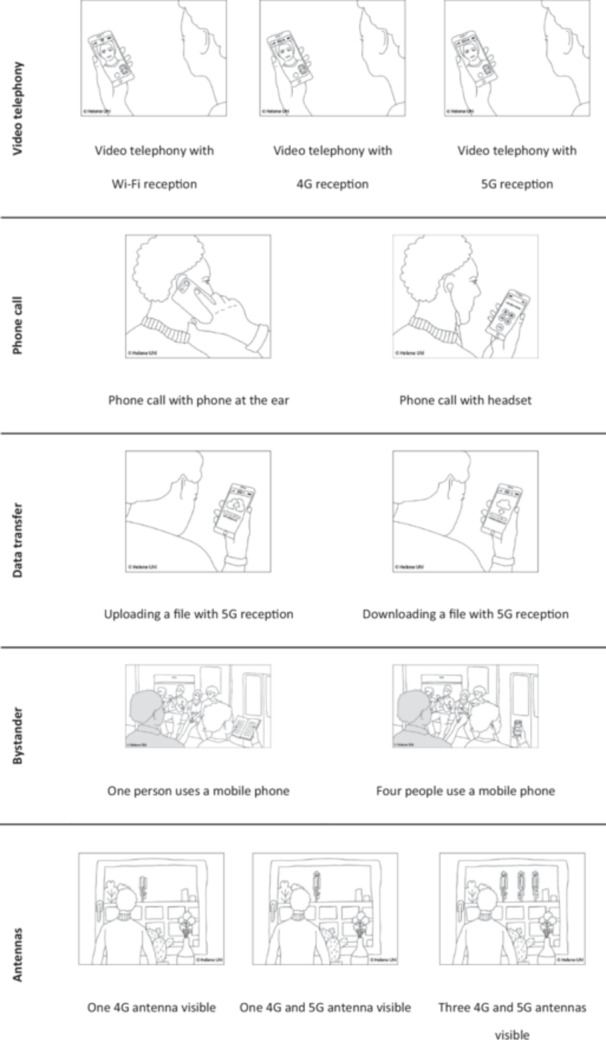
Exposure situations shown. Each row represents one page in the online version of the survey that was administered.

Situational exposure perception was assessed using professionally illustrated sketches presented in pairs or triplets on separate survey pages, differing in one exposure characteristic (network type, number of sources, proximity, or data transfer). Figure 1 shows how these situations were visualized.

A picture‐based approach was chosen because it has already been proven to be a helpful tool when assessing peoples RF‐EMF risk perception (Claassen et al. 2017; Freudenstein, Wiedemann, and Varsier 2015). However, it has only been used to assess exposure perception in a qualitative study (Link et al. 2025). Describing a situation verbally and visually gives respondents the opportunity to capture the content of a question at different levels of perception (Goldsmith 1979). By doing so, pictures can help respondents to answer survey questions in a more meaningful way, among other things, because a purely verbal description may lead to higher inter‐individual variance in the ideas of how a specific exposure situation may look like. The sketches were drawn specifically for the study by a professional illustrator. In the survey, they were displayed over the full width of the screen so that details could be seen.

In the “video telephony” situation, network type varied between 5G, 4G, and Wi‐Fi. In the “phone call” situation, the distance between the mobile phone and the head varied. For “data transfer,” the comparison was between upload and download. In the “bystander” and “antennas” situations, the number of exposure sources varied; in the latter, network type also differed (three 5G + 4G, one 5G +4 G, one 4G).

To guide respondents’ interpretations, each situation was accompanied by a short description (e.g., “The person pictured is talking to another person via video calling [4G connection].”), followed by the question: “How much do you think the person pictured is exposed to EMFs (e.g., 4G connection)?”. Responses were given on a 10‐point scale ranging from “1 =; not at all” to “10 = very much.” Real‐life situations were depicted, meaning that in some cases (e.g., the bystander scenario), more than one exposure characteristic (e.g., number and distance of sources) varied.

The order in which the five online survey pages were displayed was randomized. Further survey details are documented at https://osf.io/xbk57/?view_only=0e8b43a5f8cd49ca939b77f0402f9ef2.

### 2.4 Data Preparation and Statistical Analysis

To make the conclusions more generalizable, each participant was assigned a weight to account for deviations between the sample and the age and gender distribution in the general population of the 10 participating countries. Therefore, reported *n* values in analyses may differ slightly from the total sample (10,358). Deviations also occur because a relatively small number of respondents identified as transgender (*n* = 20), non‐binary/non‐conforming (*n* = 24) and other (*n* = 8). We considered the sample size for each of these groups too small for analysis, so all statistical models including gender as a variable only included respondents reporting binary gender (male/female).

We examined whether age, gender (binary), and education correlate with the exposure characteristics investigated. Seven paired‐sample *t*‐tests tested effects of exposure characteristics on exposure perception (Hypotheses 2–6). Differences between countries (Hypotheses 1 and RQ3) were examined with ANOVA or ANCOVA, depending on the model. To analyze differences in the evaluation of various exposure characteristics between countries, differences between the two variables of interest were calculated. For example, the value of *5G phone* minus the value of *4G phone* was calculated to examine the influence network type has. When sociodemographic variables (age, gender [binary], education) correlated with exposure characteristics, they were included as covariates. If Levene's test indicated heteroscedasticity, Welch's ANOVA was used. Post hoc comparisons between countries were performed using Tukey HSD tests where appropriate. Country differences were considered supported if at least two countries differed significantly. Analyses were conducted using SPSS version 28.0.1.0 (IBM Corp 2021).

## 3 Results

*Descriptive statistics and correlations:* Mean differences per exposure characteristic (total sample), and correlations between exposure characteristics and sociodemographic variables can be found in Table 3. Pearsons *r* is reported for age and gender (binary), and Spearman's *r* is reported for education.

**Table 3:** Correlations and mean differences between sociodemographic characteristics and situational exposure perception.

	Difference scores						
	5G minus 4G phone	5G minus 4G base station	5G minus Wi‐Fi phone	4 minus 1 bystander	3 5G minus 1 5G antenna	Phone call at ear minus headset	Upload minus download
Age	n.s.	↓**	↓**	↓**	↓*	↑**	n.s.
Gender	n.s.	n.s.	n.s.	↓**	↓**	↑**	n.s.
Education	n.s.	n.s.	n.s.	n.s.	↓*	↑**	n.s.
Mean difference [95% CI]	0.43 [0.41, 0.46]	0.49 [0.47, 0.51]	0.80 [0.77, 0.83]	1.17 [1.14, 1.20]	0.90 [0.88, 0.92]	1.31 [1.27, 1.34]	−0.08 [−0.10, −0.06]

*Note:* Gender (binary): 0 = female, 1 = male. Age categories: 16–24, 25–34, 35–44, 45–54, 55–64, 65+. Education: 1 = did not complete compulsory education, 2 = completed compulsory education, 3 = completed further education, 4 = completed university degree(s). ↑ = positive correlation, ↓ = negative correlation. Significance:

* *p* < 0.05

** *p* < 0.001, n.s. = not significant.

*General exposure perception*: Across the entire sample, respondents considered they were “moderately” exposed to RF‐EMF from mobile communications devices and base stations in their everyday life (*M* = 4.41, SD = 1.49). Country means (Table 4) ranged from *M* = 3.57 (SD = 1.56) in Finland to *M* = 4.96 (SD = 1.37) in Slovenia (distributions are presented in Supporting Information S1: Table 1). Country differences were statistically significant (*F*(9, 4095.863) = 93.938, *p* < 0.001, *η* ^*2*^ = 0.083, *f* = 0.30, *n* = 10,066). Several pairwise differences between countries were observed (see Supporting Information S1: Table 2).

**Table 4:** Means and standard deviations of everyday exposure perception and expected change in exposure.

	Everyday exposure perception		Expected change in exposure	
	*M*	SD	*M*	SD
Austria	4.65	1.39	4.91	1.07
Finland	3.57	1.56	4.36	1.10
France	4.47	1.32	4.83	1.03
Germany	4.37	1.31	4.74	1.08
Greece	4.89	1.35	4.92	1.20
Poland	3.80	1.57	4.48	1.23
Serbia	4.56	1.53	4.94	1.29
Slovenia	4.96	1.37	5.12	1.21
Spain	4.70	1.39	4.70	1.09
United Kingdom	4.11	1.48	4.67	1.02

Respondents also expected exposure levels to increase slightly with the introduction of 5G (*M* = 4.77, SD = 1.15; a mean value of 4 would indicate no change in exposure). The smallest expected increase was reported in Finland (*M* = 4.36, SD = 1.10) and the largest in Slovenia (*M* = 5.12, SD = 1.21). Means and standard deviations for all countries can be found in Table 4 (distributions are presented in Supporting Information S1: Table 3). Country differences were again statistically significant (*F*(9, 4095.36) = 38.61, *p* < 0.001, *η* ^2^ = 0.035, *f* = 0.12, *n* = 10,066). Results of the post hoc analyses are presented in Supporting Information S1: Table 4.

*Situational exposure perception*: Perceived exposure differed significantly across the examined exposure characteristics (Table 5).

**Table 5:** *T*‐test results for Hypothesis 2–6.

Condition	*M* (SD) v1	*M* (SD) v2	*t*	*df*	*p*	*d*
Network type H2A: 5G (v1) versus 4G (v2) phone	5.50 (2.43)	5.07 (2.43)	35.277	10065	< 0.001	0.35
Network type H2B: 5G (v1) versus 4G (v2) base station	5.31 (2.71)	4.82 (2.59)	43.870	10065	< 0.001	0.44
Network type H3: 5G (v1) versus Wi‐Fi (v2) phone	5.50 (2.43)	4.70 (2.45)	51.117	10065	< 0.001	0.51
Quantity H4A: 4 bystanders (v1) versus 1 bystander (v2)	5.00 (2.61)	3.83 (2.32)	76.519	10065	< 0.001	0.76
Quantity H4B: 3 5G antenna (v1) versus 1 5G antenna (v2)	6.21 (2.93)	5.21 (2.71)	76.363	10065	< 0.001	0.76
Proximity H5: Phone call at ear (v1) versus phone call with headset (v2)	6.36 (2.69)	5.05 (2.47)	77.282	10065	< 0.001	0.75
Data transfer H6: Upload (v1) versus download (v2)	4.99 (2.52)	5.07 (2.56)	−8.822	10065	< 0.001	−0.09

*Note: N* = 10,066.

Consistent with Hypotheses 2–5, both the number of exposure sources (quantity) and proximity of the mobile phone to the head (proximity) had medium‐to‐large effects on perceived exposure (*d*_quantity phone_ = 0.76, *d*_quantity base station_ = 0.76, and *d*_proximity_ = 0.75). Network type showed small‐to‐medium effects, varying by comparison: 5G/4G (*d*_phone_ = 0.35, *d*_base station_ = 0.44), or 5G/Wi‐Fi (*d* = 0.51). Concerning Hypothesis 6 we found that data transfer had a very small effect (*d* = −0.09). All comparisons were statistically significant.

The highest exposure perception was reported for a phone held to the ear (*M* = 6.36, SD = 2.69), and the lowest for the bystander situation with one third‐party phone (*M* = 3.83, SD = 2.32).

**Figure 2:**
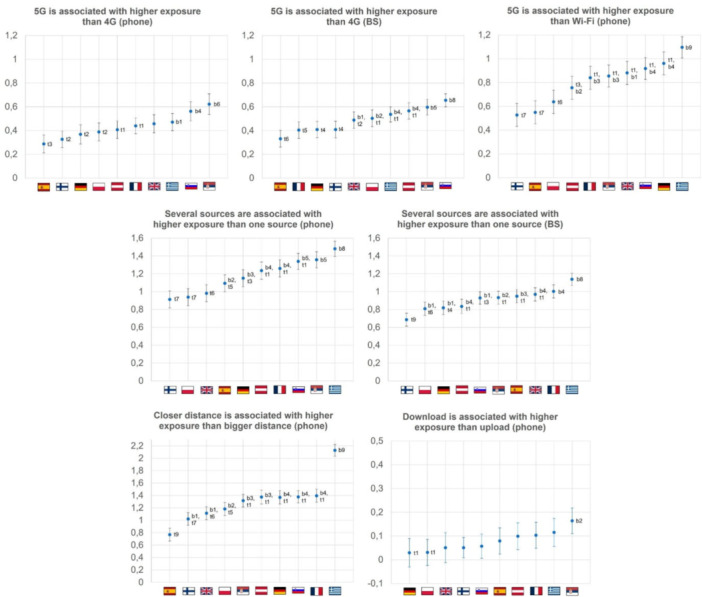
Mean differences in perceived exposure per country and comparison; “BS” short for base station. The *X*‐axis shows the countries in ascending order. The *Y*‐axis shows the difference between the two values compared, including the 95% confidence interval. BS = base station. Labeling of significant differences: t = top, b = bottom, the number refers to the number of countries that are significantly different from the top or bottom. For example, Countries with b2 differ significantly from the two countries with the lowest difference values.

*Country differences in situational exposure perception*: The mean rating for all situations (by country) can be found in Supporting Information S1: Table 5. Exposure perception differed slightly between countries across the examined exposure characteristics. Figure 2 provides an overview of the mean differences and the 95% confidence intervals for each country. However, the observed effects were small (for test statistics of the main effects see Supporting Information S1: Table 6).

*1. Network type*: Perceived exposure to 5G (vs. 4G) from mobile phones differed significantly between countries. Differences were largest in Serbia (*M* = 0.62, 95% CI [0.54, 0.71]) and smallest in Finland (*M* = 0.33, 95% CI [0.26, 0.39]).

For base stations, both country and age had significant effects, with younger respondents perceiving larger differences between 5G and 4G exposure. The perceived difference between 5G and 4G exposure was highest in Slovenia (*M* = 0.67, 95% CI [0.60, 0.73]) and lowest in Spain (*M* = 0.33, 95% CI [0.26, 0.40]).

Comparing 5G versus Wi‐Fi emissions from mobile phones, country differences were also significant. The largest differences were found in Greece (*M* = 1.10, 95% CI[1.01, 1.18]) and the smallest in Finland (*M* = 0.53, 95% CI [0.43, 0.63]). Younger respondents generally perceived greater differences than older ones.

To sum up, respondents from all 10 countries reported a higher exposure perception for 5G compared to 4G and Wi‐Fi.

*2. Number of exposure sources:* Perceived exposure increased when multiple mobile phones or base stations were present, and this effect differed slightly between countries. For mobile phones, the effect was largest in Greece (*M* = 1.48, 95% CI [1.39, 1.57]) and smallest in Finland (*M* = 0.91, 95% CI [0.82, 1.01]). Younger respondents and women perceived larger differences between one and several phones.

For base stations, country differences were also observed. Perceived exposure differences were largest in Greece (*M* = 1.14, 95% CI [1.07, 1.21]) and smallest in Finland (*M* = 0.68, 95% CI [0.61, 0.76]). Women and respondents with lower educational levels rated exposure differences higher, respectively, than men and respondents with higher educational levels.

Overall, across countries, a higher number of exposure sources was consistently associated with higher perceived exposure.

*3. Proximity of mobile phone to the head*: The perceived importance of proximity differed significantly between countries. Perceived exposure differences were highest in Greece (*M* = 2.13, 95% CI [2.03, 2.23]) and lowest in Spain (*M* = 0.77, 95% CI [0.67, 0.87]). Older respondents, men, and respondents with higher educational levels perceived larger differences in exposure.

*4. Data transfer*: Perceived exposure differed slightly between upload and download activities. The largest difference was observed in Serbia (*M* = −0.16, 95% CI [−0.22, −0.11]), and the smallest in Germany (*M* = −0.03, 95% CI [−0.89, 0.03]). Overall, the effect of data transfer on perceived exposure was very small across countries.

In summary, evaluations of exposure characteristics—network type, quantity, proximity, and data transfer—varied slightly across countries, though effect sizes were generally small (*η^2^* < 0.10; Cohen 1992). Perceptions also differed by age, gender, and educational level.

## 4 Discussion

We investigated whether respondents across 10 European countries differ in their perception of RF‐EMF exposure from mobile communication technologies and how exposure characteristics shape these perceptions. Both general and situational exposure perceptions were analyzed at the total‐sample and country levels. This study thus extends existing research by providing an overview regarding exposure perceptions from samples collected in 10 European countries.

Overall, respondents perceived their everyday exposure to RF‐EMF as moderate, yet expected it to increase with the introduction of 5G. This expectation may stem from media reporting—particularly in connection with Covid—or from heuristics such as “higher frequency/faster data transmission implies more exposure.” Moreover, new technologies often evoke initial skepticism (cf. Karahanna et al. 1999).

Situationally, the highest perceived exposure to RF‐EMF from mobile communications occurred when making a phone call with the mobile phone held to the ear, whereas the lowest perceived exposure was associated with being near one single bystander using a mobile phone. Among the four exposure characteristics examined, proximity to the head and the number of exposure sources (quantity) had the strongest influence on perceived exposure, while network type had a smaller effect and data transfer only a negligible one. A possible explanation for the minimal influence of data transfer is that people may lack a clear everyday heuristic for judging exposure in such situations, unlike for proximity and quantity. In general, 5G was perceived as causing higher exposure than 4G for both handsets and base stations, while Wi‐Fi was rated lower than both. This perception contrasts with emerging evidence suggesting that overall RF‐EMF exposure levels have changed little over the past decade and that using 5G technology may even reduce personal exposure in some situations. Likewise, situations with more exposure sources and shorter distances to the body were linked to higher perceived exposure. These findings suggest that different exposure characteristics contribute to exposure perception to varying degrees.

Freudenstein, Wiedemann, and Varsier (2015) found distance to have a stronger effect than number of sources, whereas we observed nearly identical effect sizes for proximity (*d* = 0.75) and quantity (*d* = 0.76). Unlike their study, we found the highest perceived exposure for a phone held to the ear rather than for base stations. This discrepancy may have methodological reasons, reflect increased public awareness of mobile phones as primary exposure sources (Link et al. 2025; Vaupotič et al. 2025), or indicate declining RF‐EMF risk perception over the past two decades (Boehmert 2018; Goette and Ludewig 2019, 2024).

Cross‐country differences in situational exposure perception were rather small but systematic. But given the large sample size of the present study, statistically significant differences should be interpreted carefully, since they might not necessarily reflect substantial practical differences in perception.

Proximity of the mobile phone to the head and the number of exposure sources made the biggest difference for exposure perception, followed by network type. Compared to most other countries, in Greece the influence of proximity of the mobile phone to the head was rated by far the highest, but quantity and network type also had a great influence. Extrapolating from this finding, it seems reasonable to assume that our Greek sample experiences greater variation of perceived exposure in their everyday lives. Conversely, the impact attributed to the exposure characteristics is smaller for respondents in Finland and Spain, indicating a more homogeneous exposure perception across situations, presumably also in their everyday life. Notably, in Serbia, network type had the second‐largest influence, with 5G rated higher than 4G despite 5G had not been introduced at the time of data collection, echoing findings on pre‐installation risk perception for power lines (Goette and Ludewig 2019, 2024).

To contextualize the magnitude of perceived exposure, it should be noted that the mean scores across situations ranged from 3.83 to 6.36 on the 10‐point scale. Although the scale did not include a labeled “moderate” category, responses overall fell within the mid‐range. While this study did not assess perceptions of other environmental hazards, previous research provides some context. For example, the Eurobarometer 2010 (TNS Opinion & Social 2010), Zwick (2005) and Goette and Ludewig (2019, 2024) assessed risk perceptions of hazards such as noise, air quality, water quality, food safety, mad cow disease (BSE), high voltage‐power lines, mobile communications, climate change, and vaccinations. In these comparisons, mobile communications tended to receive relatively lower concern ratings than several other, EMF‐unrelated risks. Based on these findings, it seems advisable not to overestimate perceived exposure values in the current study.

The small country effects align with the concept of intuitive toxicology (Kraus et al. 1992). People seem to rely on similar heuristics when judging the harmfulness of an agent, largely independent of cultural context. However, the remaining cross‐country variation points to the relevance of contextual and cultural factors that shape how these heuristics are applied in practice. These may include general levels of risk perception, trust in public institutions and authorities, and country‐specific communication practices related to RF‐EMF. For example, the Special Eurobarometer on EMF (TNS Opinion & Social 2010) indicates particularly high levels of concern about potential health effects of mobile communication infrastructure in Greece, which may contribute to heightened sensitivity to exposure characteristics. In addition, county differences in precautionary communication and public awareness, such as whether mobile phones are perceived as a relevant exposure source, are likely to influence how individuals interpret and evaluate exposure situations.

Although differences between countries were considerable, only a small share could be attributed to country affiliation itself, suggesting alternative explanations. Greece stood out as an outlier regarding proximity, possibly due to greater awareness of precautionary behavior, as the comparative sketch depicted headset use. This interpretation aligns with recent findings showing higher relevance of mobile communication and health issues in Greece compared to Germany (Eggeling‐Böcker et al. 2025). From a science communication perspective, these findings suggest that while general heuristics in exposure perception are broadly shared, their salience and application may differ depending on national contexts. However, intuitive rules such as “more sources imply more exposure” may not reliably reflect actual exposure patterns, given the technical complexity and context‐dependence of RF‐EMF exposure.

This implies that differences in exposure perception alone provide limited grounds for strongly tailoring communication strategies to specific countries. Instead, country‐specific adaptation may be more appropriately guided by other factors, such as differences in risk perception, trust, and existing knowledge structures.

At the same time, given the generally small magnitude of country differences observed in exposure perception, communication strategies are unlikely to require substantial country‐specific adaptation based solely on these findings. Rather, the results suggest that communication efforts may benefit more from addressing potential misconceptions regarding 5G‐related exposure. Providing clear and accessible explanations of how exposure arises may help align public perceptions more closely with current scientific evidence. In addition, communication should explicitly address potential mismatches between intuitive perceptions and technical exposure characteristics, while avoiding oversimplification and acknowledging remaining uncertainties.

Future research should examine not only individual exposure characteristics but also their complex interactions—for instance, when a person uses a headset (proximity) while others nearby use 5G (network type, quantity). It would also be valuable to explore factors shaping exposure perception beyond sociodemographic characteristics, such as cultural influences or exposure to misinformation. Given the small cross‐country differences observed, comparisons with countries where radiation protection or technology are valued differently could yield further insights.

*Limitations*. The chosen method comes with some limitations. As with any form of survey recruitment, the sample may be subject to selection bias. Participation was voluntary, and self‐selection into the survey may have influenced responses, for example, individuals with stronger interest in or opinions about RF‐EMF may have been more likely to participate. This should be considered when interpreting the results.

Moreover, observed differences between countries may partly reflect variations in response styles or cultural differences in using rating scales, rather than true differences in exposure perception.

Another limitation concerns the design of the study: With one exception, each situation varied only one exposure characteristic while keeping others constant, although real exposure results from their combined interaction. However, using sketches of realistic situations provided a more concrete operationalization than previous studies (Freudenstein, Wiedemann, and Brown 2015; Freudenstein, Wiedemann, and Varsier 2015), and this advantage outweighs the limitation of controlled variation. Also, results may have differed if comparative situations had been designed differently, for instance, if the phone in the call situation had been placed on a table or if bystanders’ devices had been positioned closer to the person depicted.

Although a broad range of exposure characteristics was examined, the comparative design allowed only a limited number of comparisons. Other factors may also influence exposure perception, but space constraints and respondent attention necessitated a focus on the four selected characteristics. Comparisons across survey pages (e.g., handset vs. base station) could not have the same level of validity, as respondents likely used within‐page situations as reference points. Nonetheless, some of these “gaps” have already been investigated in other contexts, for example, the influence of the exposure source (handset vs. base station) on perceived exposure (Cousin and Siegrist 2010b; Freudenstein, Wiedemann, and Brown 2015; Link et al. 2025).

Although the survey pages on situational exposure perception were randomized, ratings on the first page likely served as anchors for subsequent evaluations. Therefore, comparing within‐person differences (e.g., perceived exposure from 5G vs. 4G mobile phones) is more informative than absolute values. Forced‐choice questioning may also have elicited intuitive judgments even under uncertainty. Furthermore, within‐subject designs tend to overestimate effect sizes (Lakens 2013), though this does not affect comparative validity across conditions or countries.

The relationship between exposure perception and risk perception also remains unclear. The (situational) exposure perception alone does not provide information on whether mobile phone emissions are perceived as a risk in the situations investigated and whether the exposure perception of RF‐EMF is relevant in everyday life.

## 5 Conclusion

When assessing exposure to RF‐EMF, people appear to rely on simple heuristics that, in many cases, lead to reasonably accurate judgments. Their understanding of how exposure characteristics influence actual exposure can therefore be regarded as fairly adequate. At the same time, the results indicate that respondents consistently expected higher exposure from 5G compared with earlier technologies such as 4G or Wi‐Fi. While previous studies have examined exposure perception for earlier mobile communication standards in both national and international samples, comparative analyses across multiple countries have been lacking. The present study fills this gap, revealing that although country differences were generally small, they yielded valuable insights—for instance, respondents in Greece showed greater variation in perceived everyday exposure compared to other countries.

Understanding how people perceive exposure and which factors shape these perceptions is crucial for effective science communication, particularly for public health and radiation protection authorities. Identifying situations in which people feel particularly exposed, and evaluating whether these perceptions are justified can enable more targeted communication about actual exposure levels. In particular, the finding that 5G is widely perceived as increasing exposure, even though emerging evidence suggests that overall RF‐EMF exposure levels have changed little over time and may even decrease in some situations with newer technologies, highlights a potential misconception that could be addressed in risk communication. Prior research has shown that well‐designed communication can help correct misconceptions about mobile communication technologies and encourage behavioral changes to reduce exposure (Cousin et al. 2011).

## Ethics Statement

All procedures were performed in compliance with relevant laws and institutional guidelines and have been approved in the form of an ethics application (29.06.2023) by the committee of the IU International University of Applied Sciences before data collection started. The privacy of human subjects was observed and informed consent was obtained before participants began the experiment.

## Conflicts of Interest

The authors declare no conflicts of interest.

## Supporting information

Supporting File 1

Supporting File 2

Supporting File 3

## Data Availability

The data that support the findings of this study are available on request from the corresponding author. The data are not publicly available due to privacy or ethical restrictions.
