# Supplementary material for: A Ten‐Country Study on Public Perceptions of 5G EMF Emissions: Who Feels Exposed, and Why?
Source: Bioelectromagnetics. 2026 Jun 5;47(5):e70058. doi: 10.1002/bem.70058 (PMC13238347; doi:10.1002/bem.70058)
Supplement: Supplementary file 1 — Supporting File 1 [file BEM-47-0-s002.docx]

**Supplementary Materials**

Supplementary Table 1: Country differences in everyday RF-EMF exposure perception.

| Answer | Finland | Poland | UK | Germany | France | Serbia | Austria | Spain | Greece | Slovenia |
| --- | --- | --- | --- | --- | --- | --- | --- | --- | --- | --- |
| 1 | 103 (103) | 111 (113) | 41 (45) | 32 (33) | 32 (30) | 38 (38) | 25 (25) | 17 (18) | 24 (18) | 15 (14) |
| 2 | 181 (181) | 113 (117) | 111 (119) | 55 (56) | 48 (46) | 61 (63) | 46 (44) | 57 (55) | 30 (32) | 22 (23) |
| 3 | 159 (158) | 81 (84) | 117 (127) | 61 (61) | 49 (49) | 51 (53) | 57 (56) | 41 (41) | 50 (48) | 54 (55) |
| 4 | 334 (336) | 450 (469) | 433 (475) | 470 (474) | 447 (450) | 425 (439) | 393 (394) | 396 (399) | 284 (293) | 352 (344) |
| 5 | 112 (116) | 124 (129) | 134 (147) | 205 (214) | 231 (236) | 162 (167) | 223 (228) | 207 (215) | 282 (294) | 198 (202) |
| 6 | 70 (72) | 52 (54) | 81 (89) | 113 (118) | 118 (122) | 102 (103) | 144 (148) | 161 (167) | 208 (217) | 193 (201) |
| 7 | 50 (52) | 71 (74) | 91 (102) | 70 (73) | 83 (86) | 160 (176) | 122 (126) | 127 (133) | 124 (133) | 176 (185) |
| N | 1009 (1018) | 1002 (1040) | 1009 (1104) | 1007 (1029) | 1008 (1019) | 1001 (1039) | 1010 (1021) | 1007 (1029) | 1002 (1035) | 1011 (1024) |

*Note.* Question: “How much do you think you are exposed to electromagnetic fields (EMFs) from mobile communications devices and mobile phone masts (incl. 5G technology) in your everyday life?”. Scale: 1 = not at all, 4 = moderately, 7 = to a very high degree. Numbers in brackets represent the unweighted data.

Supplementary Table 2: Means, standard deviations and country differences in everyday exposure perception.

|  | Finland | Poland | UK | Germany | France | Serbia | Austria | Spain | Greece | Slovenia |
| --- | --- | --- | --- | --- | --- | --- | --- | --- | --- | --- |
| Finland | 3.57 (1.56) |  |  |  |  |  |  |  |  |  |
| Poland | ↑** | 3.80 (1.57) |  |  |  |  |  |  |  |  |
| UK | ↑** | ↑** | 4.11 (1.48) |  |  |  |  |  |  |  |
| Germany | ↑** | ↑** | ↑* | 4.37 (1.31) |  |  |  |  |  |  |
| France | ↑** | ↑** | ↑** | n.s. | 4.47 (1.32) |  |  |  |  |  |
| Serbia | ↑** | ↑** | ↑** | n.s. | n.s. | 4.56 (1.53) |  |  |  |  |
| Austria | ↑** | ↑** | ↑** | ↑** | n.s. | n.s. | 4.65 (1.39) |  |  |  |
| Spain | ↑** | ↑** | ↑** | ↑** | ↑** | n.s. | n.s. | 4.70 (1.39) |  |  |
| Greece | ↑** | ↑** | ↑** | ↑** | ↑** | ↑** | ↑* | n.s. | 4.89 (1.35) |  |
| Slovenia | ↑** | ↑** | ↑** | ↑** | ↑** | ↑** | ↑** | ↑* | n.s. | 4.96 (1.37) |

*Note.* The diagonal represents the mean values and standard deviations in everyday exposure perception for each country. The values below the diagonal are to be read as: ‘Country row’ has a higher (↑) exposure perception than ‘Country column’.
Significance: * p < .05, ** p < .001, n.s. = not significant.

Supplementary Table 3: Country differences in the changed exposure perception due to the introduction of 5G.

| Answer | Finland | Poland | UK | Spain | Germany | France | Austria | Greece | Serbia | Slovenia |
| --- | --- | --- | --- | --- | --- | --- | --- | --- | --- | --- |
| 1 | 21 (21) | 24 (25) | 5 (6) | 7 (8) | 5 (5) | 6 (6) | 2 (2) | 13 (9) | 12 (12) | 7 (7) |
| 2 | 40 (41) | 40 (42) | 14 (15) | 14 (14) | 16 (17) | 8 (8) | 9 (9) | 17 (18) | 20 (21) | 15 (16) |
| 3 | 52 (52) | 42 (44) | 35 (38) | 30 (31) | 36 (35) | 24 (25) | 23 (22) | 36 (34) | 48 (49) | 32 (32) |
| 4 | 486 (489) | 467 (485) | 437 (478) | 453 (463) | 414 (420) | 375 (380) | 367 (370) | 303 (314) | 309 (218) | 272 (277) |
| 5 | 304 (307) | 265 (275) | 356 (389) | 307 (312) | 332 (341) | 379 (382) | 359 (363) | 357 (365) | 329 (341) | 313 (310) |
| 6 | 63 (64) | 82 (84) | 89 (97) | 109 (113) | 116 (120) | 135 (137) | 138 (140) | 157 (165) | 114 (116) | 211 (215) |
| 7 | 43 (44) | 82 (85) | 73 (81) | 86 (88) | 88 (91) | 81 (81) | 112 (115) | 118 (130) | 169 (182) | 161 (167) |
| N | 1009 (1018) | 1002 (1040) | 1009 (1104) | 1007 (1029) | 1007 (1029) | 1008 (1019) | 1010 (1021) | 1002 (1035) | 1001 (1039) | 1011 (1024) |

*Note.* Question: “How much do you think people’s exposure to electromagnetic fields (EMFs) changes due to the introduction of 5G?”. Scale: 1 = decreases very much, 4 = stays the same, 7 = increases very much. Numbers in brackets represent the unweighted data.

Supplementary Table 4: Means, standard deviations and country differences in changed perception of exposure.

|  | Finland | Poland | UK | Spain | Germany | France | Austria | Greece | Serbia | Slovenia |
| --- | --- | --- | --- | --- | --- | --- | --- | --- | --- | --- |
| Finland | 4.36 (1.10) |  |  |  |  |  |  |  |  |  |
| Poland | ↑** | 4.48 (1.23) |  |  |  |  |  |  |  |  |
| UK | ↑** | ↑* | 4.67 (1.02) |  |  |  |  |  |  |  |
| Spain | ↑** | ↑** | n.s. | 4.70 (1.09) |  |  |  |  |  |  |
| Germany | ↑** | ↑** | n.s. | n.s. | 4.74 (1.08) |  |  |  |  |  |
| France | ↑** | ↑** | ↑* | n.s. | n.s. | 4.83 (1.03) |  |  |  |  |
| Austria | ↑** | ↑** | ↑** | ↑** | ↑* | n.s. | 4.91 (1.07) |  |  |  |
| Greece | ↑** | ↑** | ↑** | ↑** | ↑* | n.s. | n.s. | 4.92 (1.20) |  |  |
| Serbia | ↑** | ↑** | ↑** | ↑** | ↑* | n.s. | n.s. | n.s. | 4.94 (1.29) |  |
| Slovenia | ↑** | ↑** | ↑** | ↑** | ↑** | ↑** | ↑** | ↑** | ↑** | 5.12 (1.21) |

*Note.* The diagonal shows the mean values and standard deviations in expected change of exposure for each country. The values below the diagonal are to be read as: ‘Country row’ assumes a higher (↑) exposure due to the introduction of 5G than ‘Country column’.
Significance: * p < .05, ** p < .001, n.s. = not significant.

Supplementary Table 5: Means and standard deviations per exposure situation and country.

| **Country** | **Video telephony 4G** | **Video telephony WiFi** | **Video telephony 5G** | **One 4G Antenna** | **One 5G Antenna** | **Three 5G Antennas** | **Upload** | **Download** | **Phone call ear** | **Phone call headset** | **One bystander** | **Four bystanders** |
| --- | --- | --- | --- | --- | --- | --- | --- | --- | --- | --- | --- | --- |
| Austria | 5.43 (2.30) | 5.08 (2.35) | 5.84 (2.47) | 5.13 (2.45) | 5.70 (2.58) | 6.55 (2.75) | 5.33 (2.46) | 5.43 (2.50) | 6.77 (2.57) | 5.40 (2.39) | 4.05 (2.27) | 5.29 (2.49) |
| Finland | 3.82 (2.16) | 3.62 (2.14) | 4.15 (2.40) | 3.11 (2.09) | 3.52 (2.35) | 4.22 (2.71) | 3.81 (2.24) | 3.86 (2.29) | 4.89 (2.61) | 3.87 (2.20) | 2.85 (1.89) | 3.76 (2.28) |
| France | 5.77 (2.22) | 5.36 (2.29) | 6.21 (2.30) | 5.67 (2.34) | 6.08 (2.38) | 7.08 (2.48) | 5.68 (2.30) | 5.78 (2.34) | 7.07 (2.29) | 5.65 (2.25) | 4.49 (2.21) | 5.75 (2.39) |
| Germany | 5.31 (2.28) | 4.71 (2.34) | 5.68 (2.44) | 5.13 (2.47) | 5.54 (2.60) | 6.36 (2.79) | 5.26 (2.35) | 5.29 (2.36) | 6.67 (2.44) | 5.30 (2.29) | 4.01 (2.20) | 5.15 (2.43) |
| Greece | 5.61 (2.33) | 5.07 (2.33) | 6.08 (2.57) | 5.59 (2.58) | 6.09 (2.66) | 7.13 (2.73) | 5.36 (2.45) | 5.48 (2.50) | 7.17 (2.47) | 5.05 (2.33) | 4.14 (2.25) | 5.58 (2.57) |
| Poland | 4.06 (2.44) | 3.79 (2.41) | 4.45 (2.72) | 3.77 (2.50) | 4.28 (2.70) | 5.09 (3.01) | 4.08 (2.58) | 4.11 (2.59) | 5.34 (2.81) | 4.17 (2.49) | 3.15 (2.31) | 4.10 (2.64) |
| Serbia | 4.91 (2.53) | 4.71 (2.60) | 5.54 (2.84) | 4.74 (2.68) | 5.33 (2.88) | 6.25 (3.05) | 4.86 (2.66) | 5.02 (2.71) | 6.40 (2.81) | 5.07 (2.64) | 3.54 (2.40) | 4.89 (2.77) |
| Slovenia | 5.54 (2.37) | 5.17 (2.41) | 6.10 (2.59) | 4.83 (2.57) | 5.52 (2.71) | 6.45 (2.94) | 5.59 (2.46) | 5.65 (2.48) | 7.18 (2.56) | 5.76 (2.44) | 4.10 (2.43) | 5.43 (2.72) |
| Spain | 5.33 (2.39) | 5.06 (2.40) | 5.62 (2.54) | 5.59 (2.47) | 5.92 (2.53) | 6.86 (2.69) | 5.19 (2.45) | 5.27 (2.51) | 6.14 (2.66) | 5.37 (2.49) | 4.18 (2.30) | 5.29 (2.52) |
| UK | 4.87 (2.45) | 4.44 (2.54) | 5.33 (2.61) | 4.63 (2.46) | 5.12 (2.56) | 6.11 (2.76) | 4.75 (2.54) | 4.80 (2.60) | 5.96 (2.65) | 4.86 (2.49) | 3.77 (2.38) | 4.76 (2.54) |

Supplementary Table 6: Results of the ANOVAs and ANCOVAS on country differences in exposure perception.

| **Exposure characteristic** | **Comparison** | **Test** | **F(df1, df2)** | **p** | **η²** | **f** | **n** |
| --- | --- | --- | --- | --- | --- | --- | --- |
| Network type | 5G vs. 4G (phone) | Welch ANOVA | F(9, 4095.64) = 6.357 | < .001 | .006 | .078 | 10,066 |
| Network type | 5G vs. 4G (base station) | ANCOVA | F(9, 10595) = 2.961 | .002 | .003 | .055 | 10,615 |
| Network type | 5G vs. Wi-Fi (phone) | ANCOVA | F(9, 10595) = 3.55 | < .001 | .003 | .055 | 10,615 |
| Quantity | 4 vs. 1 bystander | ANCOVA | F(9, 10519) = 2.50 | .007 | .002 | .045 | 10,549 |
| Quantity | 3 vs. 1 base station | ANCOVA | F(9, 10509) = 4.77 | < .001 | .004 | .063 | 10,549 |
| Proximity | Phone at ear vs. headset | ANOVA | F(9, 10509) = 2.580 | .006 | .002 | .045 | 10,549 |
| Data transfer | Upload vs. download | Welch ANOVA | F(9, 4094.12) = 2.31 | .014 | .002 | .045 | 10,065 |
