# Supplementary material for: A Ten‐Country Study on Public Perceptions of 5G EMF Emissions: Who Feels Exposed, and Why?
Source: Bioelectromagnetics. 2026 Jun 5;47(5):e70058. doi: 10.1002/bem.70058 (PMC13238347; doi:10.1002/bem.70058)
Supplement: Supplementary file 2 — Supporting File 2 [file BEM-47-0-s001.docx]

**Appendix A: Deviations from preregistration**

| **#** | **Details** | | **Original Wording** | **Deviation Description** | **Reader Impact** |
| --- | --- | --- | --- | --- | --- |
| 1 | Type | Hypotheses | Hypothesis E – national differences in exposure perception and in factors influencing exposure perception  Hypothesis E1. Mean ratings of perceived everyday exposure to EMF will differ between the different countries in our sample. | Hypothesis 1A: On average, in the countries investigated, people perceive their everyday exposure to RF-EMF differently.  *PLEASE NOTE: Hypotheses E1 in the preregistration is hypothesis 1A in the paper.* | Simplification, better readability and comprehensibility |
|  | Reason | New knowledge |  |  |  |
|  | Timing | After data access |  |  |  |
| 2 | Type | Hypotheses | Hypothesis E2. Mean ratings of perceived EMF exposure change due to the introduction of 5G will differ between the different countries in our sample. | Hypothesis 1B: On average, in the countries investigated, people assume exposure to RF-EMF will increase due to the introduction of 5G to a different degree.  *PLEASE NOTE: Hypotheses E2 in the preregistration is hypothesis 1B in the paper.* | Simplification, better readability and comprehensibility |
|  | Reason | New knowledge |  |  |  |
|  | Timing | After data access |  |  |  |
| 3 | Type | Hypotheses, Analysis | Hypothesis A: 5G is associated with a higher perceived exposure than 4G and Wi-Fi.  Hypothesis A1. Mean ratings of perceived exposure are higher in the “video telephony” situation with 5G than in the “video telephony” situation with 4G.  Hypothesis A2. Mean ratings of perceived exposure are higher in the “antenna on roof” situation with the 4G and 5G antenna than with the 4G only antenna. | Hypothesis 2: On average, people think that RF-EMF emissions are higher from 5G than from 4G in terms of both mobile phone A) handsets and B) base stations.  *PLEASE NOTE: Hypotheses A1 and A2 in the preregistration are hypothesis 2 in the paper.* | Simplification, better readability and comprehensibility |
|  | Reason | New knowledge |  |  |  |
|  | Timing | After data access |  |  |  |
| 4 | Type | Hypotheses | Hypothesis A: 5G is associated with a higher perceived exposure than 4G and Wi-Fi.  Hypothesis A3. Mean ratings of perceived exposure are higher in the “video telephony” situation with 5G than in the “video telephony” situation with Wi-Fi. | Hypothesis 3: On average, people think that RF-EMF emissions from mobile phone handsets are higher from 5G than from Wi-Fi.  *PLEASE NOTE: Hypothesis A3 in the preregistration is hypothesis 3 in the paper.* | Simplification, better readability and comprehensibility |
|  | Reason | New knowledge |  |  |  |
|  | Timing | After data access |  |  |  |
| 5 | Type | Hypotheses, Analysis | Hypothesis B – quantity: A higher number of mobile phones and base stations are associated with a higher exposure perception.  Hypothesis B1. Mean ratings of perceived exposure are higher in the “train” situation with four mobile phones visible than with only one visible.  Hypothesis B2. Mean ratings of perceived exposure are higher in the “antenna on roof” situation with three 5G base stations than with one 5G base station only. | Hypothesis 4: On average, people associate a higher number of RF-EMF sources with a higher exposure perception. This is the case for mobile phone A) handsets and B) base stations.  *PLEASE NOTE: Hypotheses B1 and B2 in the preregistration are hypothesis 4 in the paper.* | Simplification, better readability and comprehensibility |
|  | Reason | New knowledge |  |  |  |
|  | Timing | After data access |  |  |  |
| 6 | Type | Hypotheses | Hypothesis C – proximity/distance: Making a phone call at the ear is associated with a higher perceived exposure than making a phone call with a headset, holding the phone in the hand.  Mean ratings of perceived exposure are higher in the “phone call” situation with the phone at the ear than in the “phone call” situation with the headset. | Hypothesis 5: On average, situations in which the mobile phone is closer to the body are associated with a higher exposure perception than situations in which the mobile phone is further away from the body.  *PLEASE NOTE: Hypothesis C in the preregistration is hypothesis 5 in the paper.* | Simplification, better readability and comprehensibility |
|  | Reason | New knowledge |  |  |  |
|  | Timing | After data access |  |  |  |
| 7 | Type | Hypotheses | Hypothesis D – data transfer: Data transfer, i.e. upload vs. download, is not associated with higher or lower levels of perceived exposure  The mean ratings of perceived exposure in the “up-/download” situation differ significantly between upload and download. | Hypothesis 6: On average, people do not differentiate between upload and download activities regarding perceived exposure.  *PLEASE NOTE: Hypothesis D in the preregistration is hypothesis 6 in the paper.* | Contradiction between sentence 1 (effect is assumed) and sentence 2 (no effect is assumed) has been resolved. In addition: Simplification, better readability and comprehensibility |
|  | Reason | Typo/Error |  |  |  |
|  | Timing | After data access |  |  |  |
| 8 | Type | Hypotheses | Hypothesis E3. The country-wise means of the difference variables (see section analysis/indices) …  E3.1 for the “videotelephony” situation  E3.2 for the “train” situation  E3.3 for the “antenna on roof” situation  E3.4 for the “phone call” situation  E3.5 for the “up-/download” situation  … differ between the different countries in our sample. | Research Question 3: Do people from different countries, age groups, genders and educational levels perceive exposure emitted by mobile communications differently?  This will be investigated for the exposure characteristics examined in Hypotheses 2-5.  *PLEASE NOTE: Hypothesis E3 in the preregistration is research question 3 in the paper.* | To find out more about who feels particularly exposed, the undirected hypothesis was transformed into a research question and expanded to include the sociodemographic characteristics age, gender and education. |
|  | Reason | Typo/Error |  |  |  |
|  | Timing | After data access |  |  |  |

**Appendix B**

Country differences in everyday RF-EMF exposure perception.

| Answer | Finland | Poland | UK | Germany | France | Serbia | Austria | Spain | Greece | Slovenia |
| --- | --- | --- | --- | --- | --- | --- | --- | --- | --- | --- |
| 1 | 103 (103) | 111 (113) | 41 (45) | 32 (33) | 32 (30) | 38 (38) | 25 (25) | 17 (18) | 24 (18) | 15 (14) |
| 2 | 181 (181) | 113 (117) | 111 (119) | 55 (56) | 48 (46) | 61 (63) | 46 (44) | 57 (55) | 30 (32) | 22 (23) |
| 3 | 159 (158) | 81 (84) | 117 (127) | 61 (61) | 49 (49) | 51 (53) | 57 (56) | 41 (41) | 50 (48) | 54 (55) |
| 4 | 334 (336) | 450 (469) | 433 (475) | 470 (474) | 447 (450) | 425 (439) | 393 (394) | 396 (399) | 284 (293) | 352 (344) |
| 5 | 112 (116) | 124 (129) | 134 (147) | 205 (214) | 231 (236) | 162 (167) | 223 (228) | 207 (215) | 282 (294) | 198 (202) |
| 6 | 70 (72) | 52 (54) | 81 (89) | 113 (118) | 118 (122) | 102 (103) | 144 (148) | 161 (167) | 208 (217) | 193 (201) |
| 7 | 50 (52) | 71 (74) | 91 (102) | 70 (73) | 83 (86) | 160 (176) | 122 (126) | 127 (133) | 124 (133) | 176 (185) |
| N | 1009 (1018) | 1002 (1040) | 1009 (1104) | 1007 (1029) | 1008 (1019) | 1001 (1039) | 1010 (1021) | 1007 (1029) | 1002 (1035) | 1011 (1024) |

*Note.* Question: “How much do you think you are exposed to electromagnetic fields (EMFs) from mobile communications devices and mobile phone masts (incl. 5G technology) in your everyday life?”. Scale: 1 = not at all, 4 = moderately, 7 = to a very high degree. Numbers in brackets represent the unweighted data.

**Appendix C**

Country differences in the changed exposure perception due to the introduction of 5G.

| Answer | Finland | Poland | UK | Spain | Germany | France | Austria | Greece | Serbia | Slovenia |
| --- | --- | --- | --- | --- | --- | --- | --- | --- | --- | --- |
| 1 | 21 (21) | 24 (25) | 5 (6) | 7 (8) | 5 (5) | 6 (6) | 2 (2) | 13 (9) | 12 (12) | 7 (7) |
| 2 | 40 (41) | 40 (42) | 14 (15) | 14 (14) | 16 (17) | 8 (8) | 9 (9) | 17 (18) | 20 (21) | 15 (16) |
| 3 | 52 (52) | 42 (44) | 35 (38) | 30 (31) | 36 (35) | 24 (25) | 23 (22) | 36 (34) | 48 (49) | 32 (32) |
| 4 | 486 (489) | 467 (485) | 437 (478) | 453 (463) | 414 (420) | 375 (380) | 367 (370) | 303 (314) | 309 (218) | 272 (277) |
| 5 | 304 (307) | 265 (275) | 356 (389) | 307 (312) | 332 (341) | 379 (382) | 359 (363) | 357 (365) | 329 (341) | 313 (310) |
| 6 | 63 (64) | 82 (84) | 89 (97) | 109 (113) | 116 (120) | 135 (137) | 138 (140) | 157 (165) | 114 (116) | 211 (215) |
| 7 | 43 (44) | 82 (85) | 73 (81) | 86 (88) | 88 (91) | 81 (81) | 112 (115) | 118 (130) | 169 (182) | 161 (167) |
| N | 1009 (1018) | 1002 (1040) | 1009 (1104) | 1007 (1029) | 1007 (1029) | 1008 (1019) | 1010 (1021) | 1002 (1035) | 1001 (1039) | 1011 (1024) |

*Note.* Question: “How much do you think people’s exposure to electromagnetic fields (EMFs) changes due to the introduction of 5G?”. Scale: 1 = decreases very much, 4 = stays the same, 7 = increases very much. Numbers in brackets represent the unweighted data.
